# Effectiveness of face masks in blocking the transmission of SARS-CoV-2: A preliminary evaluation of masks used by SARS-CoV-2-infected individuals

**DOI:** 10.1371/journal.pone.0264389

**Published:** 2022-02-23

**Authors:** Vinicius M. Mello, Cristiane M. Eller, Andreza L. Salvio, Felipe F. Nascimento, Camila M. Figueiredo, Emanuelle S. R. F. Silva, Paulo S. F. Sousa, Pamela F. Costa, Anne A. P. Paiva, Maria A. M. M. Mares-Guias, Elba R. S. Lemos, Marco A. P. Horta

**Affiliations:** 1 Viral Hepatitis Laboratory, Oswaldo Cruz Institute, Oswaldo Cruz Foundation, Rio de Janeiro, Brazil; 2 Biosafety Level 3 Facility (BSL-3), Oswaldo Cruz Foundation, Rio de Janeiro, Brazil; 3 COVID-19 Analytical Center, Oswaldo Cruz Foundation, Rio de Janeiro, Brazil; 4 Flavivirus Laboratory, Oswaldo Cruz Institute, Oswaldo Cruz Foundation, Rio de Janeiro, Brazil; 5 Hantavirosis and Rickettsiosis Laboratory, Oswaldo Cruz Institute, Oswaldo Cruz Foundation, Rio de Janeiro, Brazil; VIT University, INDIA

## Abstract

In 2019, a novel severe acute respiratory syndrome coronavirus 2 (SARS-CoV-2), which is transmitted via the airborne route, caused a new pandemic namely, “coronavirus disease 2019” (COVID-19). Although the effectiveness of face masks to prevent the transmission of SARS-CoV-2 is debated, no study has evaluated the virus-blocking efficacy of masks used by patients. We aimed to evaluate this efficacy of masks used by SARS-CoV-2-infected individuals. Data, masks used, and nasopharyngeal swab samples were obtained from these patients. Forty-five paired samples of nasopharyngeal swabs and masks were obtained and processed; the majority of masks were woven. Viral RNAs were amplified using quantitative reverse‐transcription polymerase chain reaction and detected only on the inner parts of masks. Median viral load (VL) values of swabs and masks were 1.954x10^6^ and 2,51x10^3^, respectively. Statistically, there was a difference of approximately 1000 RNA copies/mL between swabs and masks and no significant difference in VL values among different types of masks. There were statistically significant differences in VL values between men and women and between symptomatic and asymptomatic patients. Our findings suggest the blocking of virus transmission by different types of masks and reinforce the use of masks by both infected and non-infected individuals.

## 1. Introduction

In 2019, a new respiratory coronavirus named, severe acute respiratory syndrome coronavirus 2 (SARS-CoV-2), which is transmitted via the airborne route, primarily through respiratory droplets and aerosols, caused the new global pandemic. This new virus was associated with a respiratory syndrome denominated as “coronavirus disease 2019” (COVID-19) that has resulted in millions of deaths [1, 2]. Some studies suggest that the use of a mask can potentially prevent the transmission of several respiratory viruses, such as influenza and rhinovirus, in addition to the new coronavirus [3–5]. Although there has been much discussion regarding whether masks should be used to prevent viral transmission during the initial period of the COVID-19 pandemic, there is now a global understanding of the importance of using masks for preventing SARS-CoV-2 infection. It has been reported that masks not only protect the person who is wearing it, but also reduce the likelihood of disease transmission from the person wearing the mask to another person [6].

Current epidemiological data indicate that wearing a mask can reduce the emission of SARS-CoV-2 particles into the environment [7]. The surgical mask (non-woven mask) had a greater filtration efficiency for viral aerosols; however, the filtration efficiency was inferior to that of an N95 mask [8–11]. With the worsening of the pandemic in some countries, especially the developing ones, countries have suffered from the non-availability of surgical masks [8, 12, 13]. As a great alternative, homemade fabric masks have become very popular in several affected countries, mainly in Brazil [14–20]. Although fabric masks provide less protection and have a low filtering efficiency when compared with surgical masks, they may have some effectiveness in preventing the transmission of SARS-CoV-2 [8–11]. Nevertheless, these homemade masks are produced by small-scale fashion productions and do not have quality certifications from health authorities [14–20].

Despite the World Health Organization recommendations about the use of face masks, whether it reduces the risk of transmission of SARS-CoV-2 is still controversial [21]. Few or no studies evaluated the presence of retained viruses on the masks of different materials, as well as the effectiveness of these masks in preventing viral transmission. Considering the heterogeneity of cloth masks that are sold in Brazil, it is still unclear whether these homemade masks are effective in blocking the transmission of the virus. Considering these points, in the present study, we aimed to evaluate the virus-blocking efficacy of masks used by SARS-CoV-2-infected individuals.

The results presented here suggest that the use of masks helps to block viral trans-mission by SARS-CoV-2-infected individuals and reinforce the importance of using masks as a preventive measure against the viral transmission.

## 2. Materials and methods

### 2.1. Ethics statement

The Oswaldo Cruz Institute/IOC/FIOCRUZ Research Ethics Committee approved this study (number: CAAE 37142520.0.0000.5248). All procedures were performed in accordance with the ethical standards of the responsible committees on human experimentation (institutional and national) and the Helsinki Declaration of 1975, as revised in 2008. All patients who were included in the study agreed to their participation in the research by signing the informed consent form.

### 2.2. Study population and sample collection

Nasopharyngeal swab samples and masks were collected (between December 2020 to March 2021) from patients who were suspected to be infected by SARS-CoV-2 and attended the Municipal Theatre and Benjamin Constant Institute survey, conducted in the city of Rio de Janeiro, Brazil, according to medical decision and after obtaining permissions from the patients.

Samples were collected as follows: a nasopharyngeal swab was inserted in the nostril until it hit an obstacle (the inferior concha or the back of the nasopharyngeal cavity), rotated, and removed. The test was conducted in two nostrils per patient. After sampling, the nasopharyngeal swab was inserted into a vial containing 3 mL of a viral transport medium (VTM; Xpert nasopharyngeal sample collection kit, Cepheid, Sunnyvale, CA, USA). After the collection of swab samples, the masks used for 2–3 h by the participants were placed inside a clean plastic bag and they were provided clean, new masks for use. Furthermore, data, including the biological sex and age of these patients were collected.

### 2.3. Processing of masks and swabs

**Fig 1:**
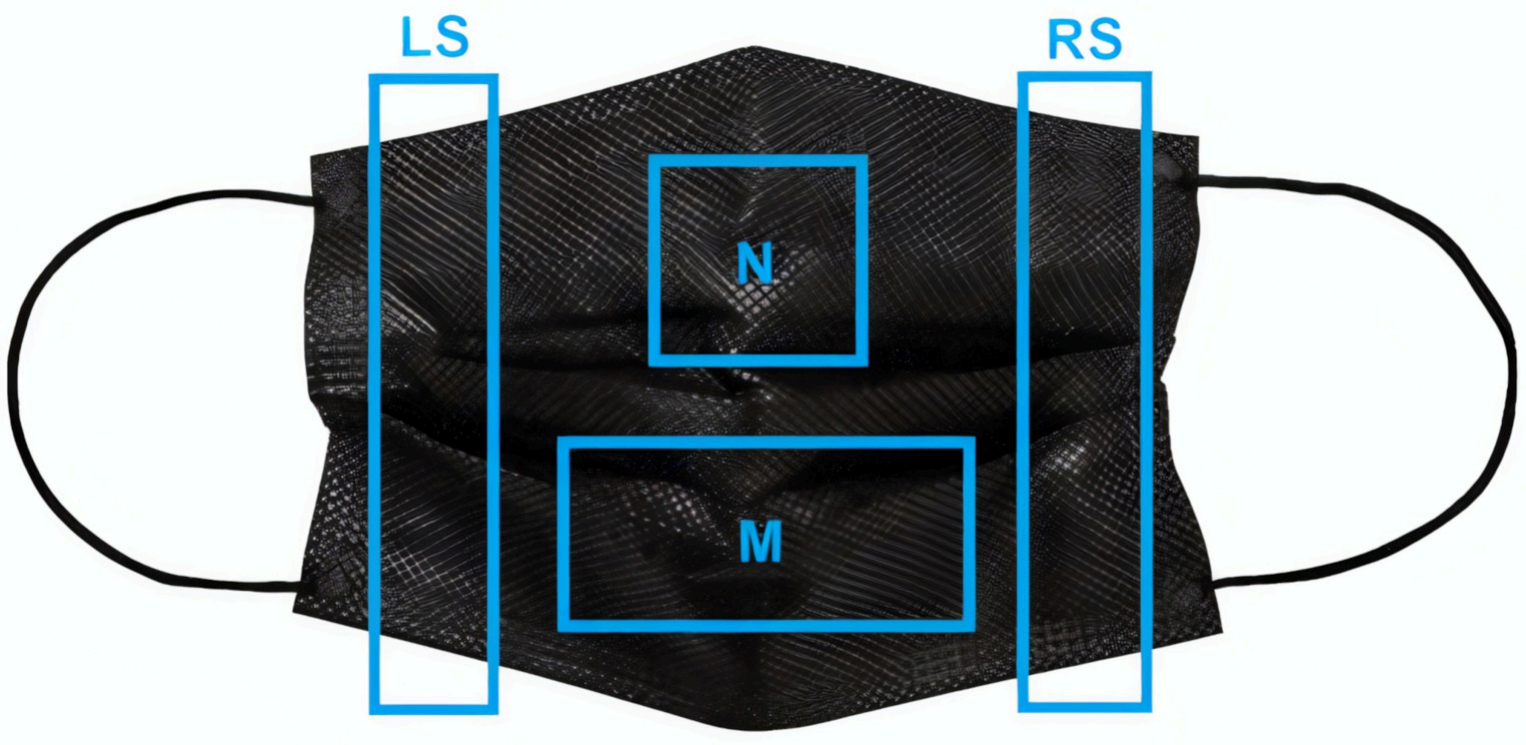
The scheme for cutting a mask. The areas enclosed within blue margins represent the areas cut from the mask. LS = Left Side, RS = Right Side, M = Mouth area, and N = Nose area.

The nasopharyngeal swab specimen was collected and immediately resuspended in 3 mL of the VTM. For mask samples, immediately after the collection of masks, pieces were cut based on the following reference measures: the right side and left side areas with a width of 2 cm each, obtained after removing side seam using the entire height of the mask; the nose area (N) with a height of 5 cm and width of 5 cm; and the mouth area (M) with a height of 5 cm and width of 8 cm, and subsequently, these pieces were added to the VTM. In cases of samples with double or triple layers of the material, these areas were subdivided into inner part of N, middle part of N, outer part of N, inner part of M, middle part of M, and outside part of M, respecting the sizes of the cut areas previously described **(Fig 1)**. The parts with the lowest CT among the four main parts of the masks (mouth, nose, left side and right side) were selected for analysis, the table presents only this result. At this first moment, we hadn’t analyzed the middle layer (this layer is located between the inner and outer layers in non-woven masks that have three layers of protection).

Between resuspension and the processing of each sample (swabs and mask pieces), incubation at 4°C for a minimum of 30 minutes and a maximum of 12 hours was be performed. Subsequently, the samples were processed through vortex homogenization and transferred from the medium to a previously identified 1.5-mL tube using a Pasteur pipette (2 mL). Then, swabs and masks were discarded, and the final sample in the medium was stored at -80°C.

### 2.4. Viral genome extraction

Nucleic acid from all the samples was extracted and purified using the DNA/RNA 300 kit H96 in the Janus G3 and Janus Chemagic automatic extractor (Perkin-Elmer, Waltham, USA). The Janus 360 system is based on magnetic spheres for extracting viral nucleic acids from 300 uL of the sample. The operation of the equipment and the use of the commercial kit were in accordance with the manufacturer’s instructions.

### 2.5. SARS-CoV-2 molecular detection

For SARS-CoV-2 genome amplification, we used a molecular kit for the E region (Bio-Manguinhos, Rio de Janeiro, BR) following the manufacturer’s instructions. The plate setup was automated and performed using Janus G3 (Perkin-Elmer, Waltham, USA). In this method, the quantitative reverse‐transcription polymerase chain reaction (qRT-PCR) also allowed the quantification of viral genomic RNA of SARS-CoV-2 with the application of an in-house ssRNA standard curve. The chosen commercial kit helped in detecting the E region of the genome using a FAM probe and the RP human gene using a VIC probe; the latter functions as the internal positive control of the assay. For all assays, positive and negative controls were included in the commercial molecular kit, and they were used in all experiments.

Samples with a cycle threshold (Ct) value lower than 38.0 for E region were considered positive, and negative samples were the ones that presented a Ct value higher than 38.0 or no Ct value at all. For the RP target, a Ct value equal to or lower than 35.0 validated the experiment. The positive control Ct value must be lower than 37.0 to validate the assay. All samples that that presented a higher cycle threshold (CT ± 38) and that had viral load, had the qPCR repeated for confirmation, those which had detectable RNA copies in both analyses were considered positive.

### 2.6. Statistical analysis

The results of descriptive statistical analyses are presented using frequency tabulations and percentages. Medians are presented with interquartile range (IQR) values. The Mann–Whitney U test was used to compare the differences in viral loads between the independent groups of masks and swabs. Statistical significance was set at a p-value *≤ 0*.*05*. All analyses were performed using R software version 4.1.0 (The R Foundation for Statistical Computing, Vienna, Austria).

## 3. Results

Forty-five swab samples with their paired respective masks were collected. The masks were classified as woven masks (30/45; 66.7%) and surgical non-woven masks (15/45, 33.3%). SARS-CoV-2 RNA was detected in all swab samples and 24/45 (53,3%) inner part of the masks (CTs <38). One/24 mask with Ct > 38 (Ct 38.02) was considered positive after having the qPCR repeated for confirmation, having a detectable viral load of 2.39x10^3^ copies/mL. The viral RNA was detected only on the inner part (the part that was in contact with the face) of the masks. None of the masks was positive for the RNA on the outer part (the part that was in contact with the external environment). Through qPCR, we observed that 7/24 (29,1%) masks had positive CT in the left and right sides, these CTs were all above 25, ranging from 25 to 38. The median viral load values of the swab and mask samples were 1.954x10^6^ (IQR, 1.91x10^5^–2.34x10^8^) and 2,51x10^3^ (IQR, 0.0–2,51x10^3^), respectively. The descriptive information can be seen in **Table 1**.

**Table 1:** Epidemiological characteristics of patients infected with SARS–CoV–2 in the present study.

Sample	Age Range	Sex	Swab Ct	Swab VL	Mask Ct	Mask VL	Material	Symptoms
**1**	20 to 29 years	Male	23.97	4.33x10^7^	36.65	6.21x10^3^	Surgical	Yes
**2**	20 to 29 years	Male	31.47	2.30x10^5^	37.95	2.51x10^3^	Surgical	Yes
**3**	20 to 29 years	Male	34.18	3.48x10^4^	38.02	2.39x10^3^	Surgical	Yes
**4**	20 to 29 years	Male	31.43	2.37x10^5^	40.0	0	Surgical	Yes
**5**	40 to 49 years	Male	20.34	5.46x10^8^	26.52	7.31x10^6^	Woven	No
**6**	60 to 69 years	Female	20.75	4.10x10^8^	24.6	2.79x10^7^	Woven	Yes
**7**	60 to 69 years	Male	32.81	9.06x10^4^	40.0	0	Woven	No
**8**	40 to 49 years	Male	35.79	1.13x10^4^	40.0	0	Woven	Yes
**9**	40 to 49 years	Male	16.17	1.00x10^10^	30.59	4.27x10^5^	Woven	Yes
**10**	20 to 29 years	Female	24.48	3.03x10^7^	34.74	2.36x10^4^	Woven	Yes
**11**	30 to 39 years	Male	30.26	5.37x10^5^	40.0	0	Woven	Yes
**12**	60 to 69 years	Male	28.06	2.49x10^6^	40.0	0	Surgical	Yes
**13**	30 to 39 years	Female	21.34	2.71x10^8^	28.99	1.30x10^6^	Woven	Yes
**14**	60 to 69 years	Female	31.05	3.09x10^5^	40.0	0	Woven	No
**15**	40 to 49 years	Female	20.11	6.41x10^8^	34.08	3.73x10^4^	Woven	Yes
**16**	40 to 49 years	Female	32.6	1.04x10^5^	35.18	1.73x10^4^	Woven	Yes
**17**	60 to 69 years	Female	16.0	1.12x10^10^	25.11	1.96x10^7^	Woven	Yes
**18**	20 to 29 years	Male	21.55	2.34x10^8^	37.71	2.96x10^3^	Surgical	Yes
**19**	60 to 69 years	Male	15.49	1.61x10^10^	40.0	0	Woven	No
**20**	20 to 29 years	Female	16.07	1.07x10^10^	28.9	1.39x10^6^	Woven	Yes
**21**	30 to 39 years	Male	20.54	4.74x10^8^	33.5	5.60x10^4^	Woven	Yes
**22**	70 years or more	Male	33.84	4.41x10^4^	40.0	0	Woven	No
**23**	40 to 49 years	Male	34.9	2.10x10^4^	40.0	0	Woven	No
**24**	50 to 59 years	Female	24.87	2.31x10^7^	40.0	0	Woven	Yes
**25**	50 to 59 years	Female	24.87	2.31x10^7^	40.0	0	Woven	Yes
**26**	50 to 59 years	Female	24.87	2.31x10^7^	24.05	4.10x10^7^	Woven	Yes
**27**	50 to 59 years	Male	31.74	1.91x10^5^	40.0	0	Woven	Yes
**28**	50 to 59 years	Male	31.74	1.91x10^5^	36.58	6.52x10^3^	Woven	Yes
**29**	50 to 59 years	Male	31.74	1.91x10^5^	37.06	4.66x10^3^	Woven	Yes
**30**	20 to 29 years	Female	15.13	2.07x10^10^	25.61	1.38x10^7^	Surgical	Yes
**31**	50 to 59 years	Male	37.37	3.75x10^3^	40.0	0	Woven	No
**32**	50 to 59 years	Female	35.29	1.60x10^4^	40.0	0	Woven	Yes
**33**	50 to 59 years	Female	37.28	4.00x10^3^	40.0	0	Woven	No
**34**	30 to 39 years	Male	30.52	4.48x10^5^	40.0	0	Surgical	Yes
**35**	30 to 39 years	Female	28.41	1.95x10^6^	34.34	3.11x10^4^	Surgical	Yes
**36**	50 to 59 years	Female	24.87	2.31x10^7^	29.65	8.22x10^5^	Woven	Yes
**37**	20 to 29 years	Female	28.58	1.73x10^6^	40.0	0	Surgical	Yes
**38**	40 to 49 years	Male	23.79	4.91x10^7^	31.13	2.93x10^5^	Woven	No
**39**	60 to 69 years	Male	32.84	8.87x10^4^	40.0	0	Surgical	No
**40**	20 to 29 years	Female	28.04	2.52x10^6^	40.0	0	Surgical	Yes
**41**	30 to 39 years	Male	31.72	1.93x10^5^	40.0	0	Surgical	No
**42**	30 to 39 years	Male	30.7	3.95x10^5^	37.46	3.53x10^3^	Surgical	No
**43**	50 to 59 years	Male	36.78	5.67x10^3^	40.0	0	Surgical	No
**44**	30 to 39 years	Female	24.17	3.76x10^7^	35.44	1.45x10^4^	Woven	Yes
**45**	30 to 39 years	Female	14.39	3.47x10^10^	29.93	6.76x10^5^	Woven	Yes

Ct = cycle threshold; VL = Viral Load (copies/mL).

**Fig 2:**
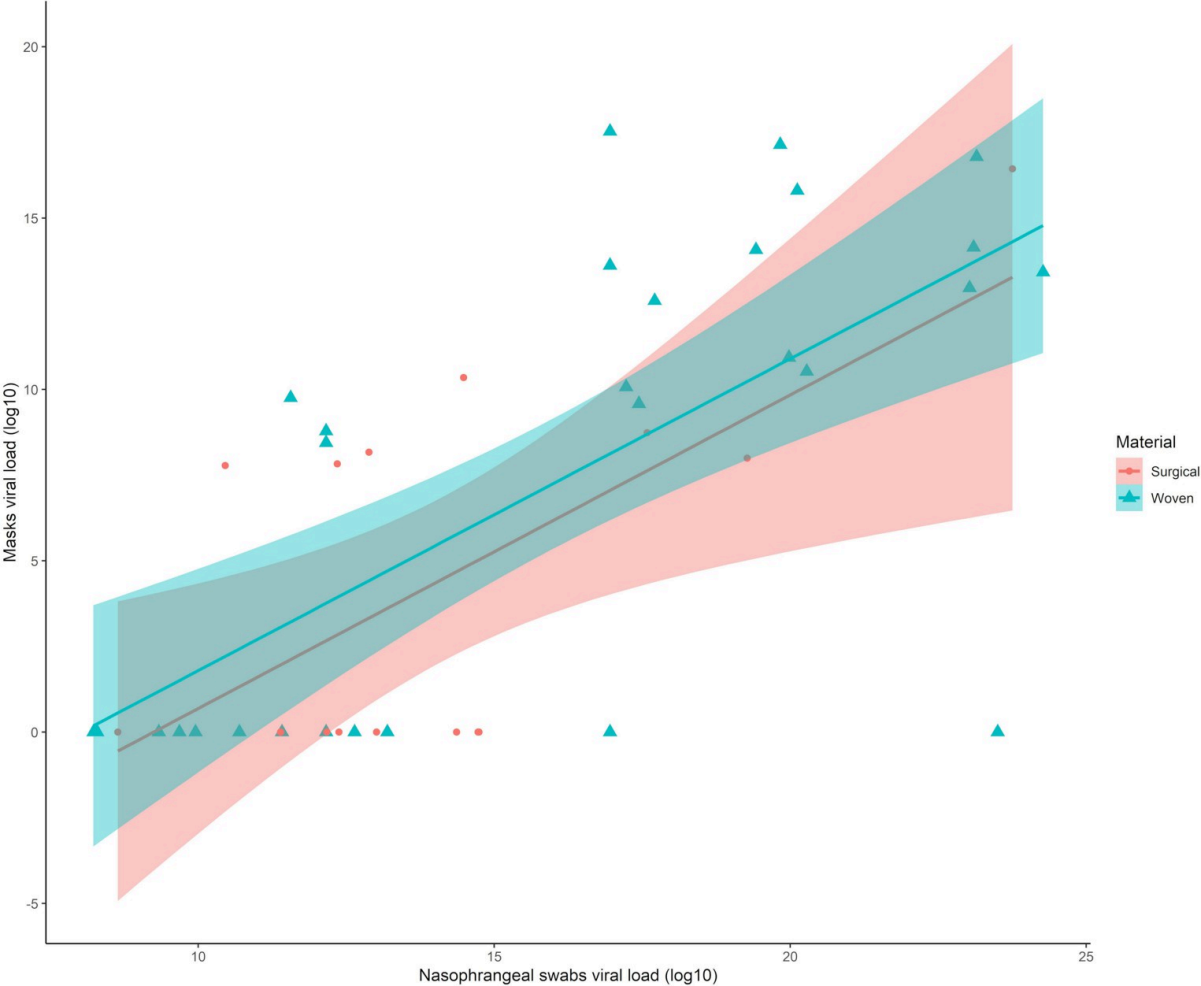
Relationship between severe acute respiratory syndrome coronavirus 2 viral loads of nasopharyngeal swabs and masks used by infected patients.

Our analysis showed a reduction of approximately ≅3 logs or 1000 RNA copies/mL (≅10 Ct values) between swab and mask samples. Statistical analysis, considering the adjusted linear equation showed a relationship in the reduction of viral load of nasopharyngeal swabs and masks (Y = -7.99 + 0.93X) with a positive and significant correlation (rho = 0.67, *p<0*.*001*) (**Fig 2**).

The analysis did not identify a statistically significant difference in median viral load values between surgical and cloth masks (U = 163, *p = 0*.*11*). The same result was obtained when comparing Ct values of nasopharyngeal swabs (U = 190, *p = 0*.*40*). Viral load was significantly higher in men than in women for masks (U = 350, *p = 0*.*01*) and swabs (U = 349, *p = 0*.*02*). We observed viral load values were significantly higher in asymptomatic than in symptomatic patients (U = 149, *p = 0*.*03*). Further results of statistical analysis can be found in **Table 2**.

**Table 2:** Median viral load values of masks and nasopharyngeal swabs from 45 patients infected by SARS–CoV–2.

	N (%)	Masks VL	IQR	*P-value*	Swab VL	IQR	*P-value*
**Sex**				*0*.*01*			*0*.*02*
*Female*	20 (44.4)	2.73x10^4^	(0.0–1.32x10^6^)		2.31x10^7^	(1.89x10^6^–4.67x10^8^)	
*Male*	25 (55.6)	0.0	(0.0–4.66x10^3^)		2.30x10^5^	(8.87x10^4^–4.33x10^7^)	
**Mask type**				0.11			0.40
*Surgical*	15 (33.3)	0.0	(0.0–3.24x10^3^)		4.48x10^5^	(2.12x10^5^–2.51x10^6^)	
*Woven*	30 (66.7)	1.05x10^4^	(6.13x10^5^–4.10x10^7^)		2.31x10^7^	(1.26x10^5^–4.58x10^8^)	
**Symptoms**				*0*.*03*			*0*.*01*
*Yes*	32 (71.1)	6.21x10^3^	(0.0–6.97x10^3^)		2.31x10^7^	(4.48x10^5^–2.71x10^8^)	
*No*	13 (28.9)	0.0	(0.0–6.76x10^5^)		9.77x10^4^	(1.86x10^4^–1.25x10^7^)	

IQR = Interquartile range; VL = Viral Load (copies/mL).

## 4. Discussion

One year after the COVID-19 pandemic, the Americas have become the epicenter of COVID-19 cases and deaths, especially in Brazil, there has been an increase in the average number of deaths [1]. This may be associated with late interventions against the pandemic and adherence to scientific negationism, for example, not wearing protective masks, among other factors [22, 23].

The results of this study reinforce the evidence that in general, wearing masks can be beneficial to the community and that this beneficial effect is derived from the ability of masks to block the exhalation and inhalation of infectious viruses, regardless of the type of mask used, as shown in a review by Brooks and Butler (2021) [24].

Data from different studies conducted in several countries have shown that the use of masks together with social distancing can reduce the transmission of SARS-CoV-2 and the number of cases of SARS-CoV-2 infection [11, 25–28]. A study performed by Ma et al (2020), which used an automated system that mimicked human breathing, showed that the virus-blocking rates of surgical and homemade masks were approximately 97% and 95%. Respectively [29]. Another study performed by Morais et al (2021), which used a similar methodology for evaluating different mask types, demonstrated similar results, where surgical masks had a filtration rate of 89% and homemade masks had filtration rates ranging from 40% to 83%, depending on the type of the fabric [30]. Although these studies show promising results, it should be noted, that in both cases the masks were sealed to the test apparatus and that the studies therefore did not include the effects of aerosol leakage through face seal leaks (gaps between the mask and the face), which can occur in a real clinical setting.

In contrast to other studies, Lindsley and colleagues (2021), in addition to evaluating the masks in an automated system, also evaluated the fit of the ones to the face of individuals. Despite presenting similar results to the others, regarding filtering and blocking, it was observed that these factors can be affected when related the fit of the masks to the face of the individual is taken into consideration [31]. The hypothesis of facial fit and incorrect handling of the mask may possibly answer and be related to the fact that some masks (7/24, 29.1%) in our study presented positive inner sides.

In the present study we observed a reduction by approximately ≅ 3 logs or ≅ 1000 RNA copies/mL (10 Ct values) for masks compared with the paired swabs collected from the same individual. These findings corroborate with data from the previous studies [29, 30, 32] that indicate a possible blocking of viral transmission by masks worn by infected people; these results may shed light on the effectiveness of masks in blocking SARS-CoV-2.

Another result that reinforced the hypothesis of blocking of viral transmission by masks was that only inner parts (the parts in contact with the face) of the masks were positive for viral RNA. Furthermore, the reduction in viral load of nasopharyngeal swabs and masks showed a significant statistical association (rho = 0.67, *p<0*.*001*), showing that the virus-blocking rate is possibly relevant in preventing the transmission of the virus from infected people to other individuals, corroborating the results found in the literature [24].

It is important to highlight that a reduction in viral load was observed in the different types of masks (non-woven and woven masks) analyzed, upon comparing swab samples and masks, which were collected simultaneously. Additionally, there was no statistically significant difference in the decrease in viral load among the different types of masks. These results reveal that different types of masks may be used to reduce the transmission of viruses to the environment and prevent infection in susceptible individuals. A similar result was obtained in a study performed by Zangmeister et al (2020) that evaluated the effectiveness of the materials of cloth masks, which were used to reduce the transmission of SARS-CoV-2, in the filtration of nanoscale aerosols and showed that and found that cloth masks did not perform similar to an N95 mask. However, woven and non-woven cloth masks may be used to reduce the transmission of SARS-CoV-2 and to filter viral particles [33].

In this context, in a country like Brazil, where it is impossible to totally adopt measures of social distancing, mainly in socially vulnerable populations in peripheral areas and slums, the use of masks seems essential. Moreover, the use of masks could be beneficial to those individuals who still need to use public transport, such as buses, trains, and/or subways, which are often crowded [21, 34–36]. The use of masks, especially woven ones, is extremely relevant as an additional protective measure for reducing the increasing number of cases and deaths due to COVID-19 in Brazil [12].

Statistically significant results were obtained on comparing viral load for swabs and masks (p = *0*.*01* and p = *0*.*02*, respectively) between men and women. This may be directly associated with the sex a hypothesis to be considered is a greater release of viral particles by males. Some studies have shown that males have a significantly high risk of severe disease, mainly due to differences in inflammatory responses to viral infections. and genetic and hormonal regulation [37–39]. However, more studies are needed to understand the underlying biological phenomena.

Some studies suggest that the viral load found in asymptomatic patients is similar to that found in symptomatic patients [40–42]. However, we identified lower Cts values in symptomatic patients than those in asymptomatic patients, and this difference was statistically significant (*p = 0*.*01*), indicating an elevated viral load mainly in swab samples **(Table 2)**.

This was a preliminary study and has some limitations. The sample size was relatively small, and this study did not evaluate the filtering efficiency of the masks as performed in some other studies [28, 29]. Additionally, it was not yet possible to assess the number of viral particles retained and recovered from the masks, nor the consistency of the extraction methodology. However, we recognize that these experiments would be difficult to carry out in a clinical setting. Furthermore, this study only evaluated masks from SARS-CoV-2 infected individuals with a positive qRT-PCRA. Further studies are needed to evaluate the masks of uninfected individuals who have had direct contact with infected individuals. Further studies. including a larger number of masks, are also needed to analyze the viability of the virus detected in infected masks through cell culture.

Nevertheless, our results provided real-life evidence regarding blocking of viral transmission by masks used by individuals infected by SARS-CoV-2. Furthermore, the results also reinforce the suggestion to use a mask by everyone, regardless of whether the individual is infected or not. This is important since there are asymptomatic cases of infection and evidence of virus transmission even before the appearance of the first symptoms [40, 43].

## 5. Conclusions

The study results shed light on the importance of using masks by individuals infected with SARS-CoV-2 and show that different types of masks can help block viral transmission. Moreover, our findings also reinforce the importance of using masks as a preventive measure against the viral transmission, regardless of the type of mask used, in addition to social distancing and personal hygiene measures.
